# Effects of high-definition transcranial direct current stimulation on the cortical−muscular functional coupling and muscular activities of ankle dorsi−plantarflexion under running-induced fatigue

**DOI:** 10.3389/fphys.2023.1263309

**Published:** 2023-09-29

**Authors:** Jianglong Zhan, Changxiao Yu, Songlin Xiao, Bin Shen, Chuyi Zhang, Junhong Zhou, Weijie Fu

**Affiliations:** ^1^ Key Laboratory of Exercise and Health Sciences of Ministry of Education, School of Exercise and Health, Shanghai University of Sport, Shanghai, China; ^2^ The Hinda and Arthur Marcus Institute for Aging Research, Hebrew SeniorLife, Boston, MA, United States; ^3^ Harvard Medical School, Boston, MA, United States

**Keywords:** exercise-induced fatigue, corticomuscular coherence, corticospinal pathway, transcranial electrical stimulation, muscle activation

## Abstract

Transcranial direct current stimulation (tDCS) can improve motor control performance under fatigue. However, the influences of tDCS on factors contributing to motor control (e.g., cortical−muscular functional coupling, CMFC) are unclear. This double-blinded and randomized study examined the effects of high-definition tDCS (HD-tDCS) on muscular activities of dorsiflexors and plantarflexors and CMFC when performing ankle dorsi–plantarflexion under fatigue. Twenty-four male adults were randomly assigned to receive five sessions of 20-min HD-tDCS targeting primary motor cortex (M1) or sham stimulation. Three days before and 1 day after the intervention, participants completed ankle dorsi–plantarflexion under fatigue induced by prolonged running exercise. During the task, electroencephalography (EEG) of M1 (e.g., C1, Cz) and surface electromyography (sEMG) of several muscles (e.g., tibialis anterior [TA]) were recorded synchronously. The corticomuscular coherence (CMC), root mean square (RMS) of sEMG, blood lactate, and maximal voluntary isometric contraction (MVC) of ankle dorsiflexors and plantarflexors were obtained. Before stimulation, greater beta- and gamma-band CMC between M1 and TA were significantly associated with greater RMS of TA (*r* = 0.460–0.619, *p* = 0.001–0.024). The beta- and gamma-band CMC of C1-TA and Cz-TA, and RMS of TA and MVC torque of dorsiflexors were significantly higher after HD-tDCS than those at pre-intervention in the HD-tDCS group and post-intervention in the control group (*p* = 0.002–0.046). However, the HD-tDCS-induced changes in CMC and muscle activities were not significantly associated (*r* = 0.050–0.128, *p* = 0.693–0.878). HD-tDCS applied over M1 can enhance the muscular activities of ankle dorsiflexion under fatigue and related CMFC.

## 1 Introduction

Long-term and high-intensity running can induce neuromuscular fatigue of lower extremities (e.g., ankle joint) (Kulmala et al., 2016), leading to task failure and sports injuries (Millet and Lepers, 2004; Giandolini et al., 2016). The decreased motor control performance under running-induced fatigue is closely associated with a decrease in neural drive that the motor cortex of the brain sends to muscles (Millet and Lepers, 2004; Girard et al., 2012), reducing the functional connectivity between the supraspinal networks and muscles, which can be captured by diminished cortical–muscular functional coupling (CMFC) (Kilner et al., 2000; Marsden et al., 2000; Liu J. et al., 2019). Previous studies showed that the decrease of CMFC, which is oftentimes assessed by corticomuscular coherence (CMC) (Grosse et al., 2002), was associated with the deepening of fatigue (Yang et al., 2009; Siemionow et al., 2010; Tuncel et al., 2010), leading to the decline in neural transmission efficiency and increased instability of neuromuscular connections, affecting motor control performance (Gandevia, 2001; Hug et al., 2015). Therefore, strategies with the goal to enhance CMFC may help improve motor control performance under fatigue and ultimately prevent fatigue-related injuries in exercise.

High-definition transcranial direct current stimulation (HD-tDCS) is one such kind of strategy that can safely increase cortical excitability (Amann et al., 2022) and neural drive to target muscles (Etemadi et al., 2023). TDCS have been used as a non-invasive brain stimulation technique to enhance cognitive functions, improve motor performance, and alleviate fatigue in humans (Amann et al., 2022). It modulates the excitability of target brain regions through the application of low-intensity electrical currents via the anode and cathode electrodes placed on the scalp. HD-tDCS can reduce current diffusion that is oftentimes observed in traditional large sponge-based tDCS, providing more focused and longer-lasting modulation of brain activity (Minhas et al., 2010; Kuo et al., 2013). A previous study, for example, observed that tDCS applied over the primary motor cortex (M1) could extend the time to exhaustion and improve the maximum torque during an isometric fatiguing elbow flexion task by enhancing neural drive to muscles (Wang et al., 2022). Moreover, tDCS over M1 could enhance the endurance performance in multi-joint coordination tasks (Liu X. et al., 2019) and reduce the perception of fatigue (Angius et al., 2018; Angius et al., 2019) by increasing the regional or whole-brain functional connectivity. However, the effects of tDCS on CMFC have not been explicitly examined. Further exploration in this subject may help better elucidate the mechanisms of the benefits of tDCS for motor control under fatigue and its related consequences.

In this study, we explored the effects of five sessions of HD-tDCS on CMFC and muscular activities of dorsiflexors and plantarflexors during ankle dorsi–plantarflexion under fatigue induced by prolonged running. We hypothesized that in a group of recreational younger adults, HD-tDCS would enhance CMFC and increase the muscular activities of dorsiflexors and plantarflexors under fatigue compared with the control (i.e., sham stimulation).

## 2 Materials and methods

### 2.1 Participants

The sample size for this study was estimated based on a previous study that utilized HD-tDCS on chronic stroke participants with the same outcome variable (i.e., CMC) (Bao et al., 2019). The value of the effect size ( $\eta_{p}^{2}=0.191$ ) was observed in the previous study, which is equal to the effect size value of f (f = 0.56). Considering the differences between healthy and stroke participants (i.e., the modulatory effect of tDCS may be weaker in healthy participants) (Hendy and Kidgell, 2013), there is a conservative reduction in effect size in the current study. Thus, the sample size was calculated using G*Power 3.1.9.7 software (Franz Faul, University of Kiel, Kiel, Germany) with a statistical power of 0.95, a probability level of 0.05, and an effect size of 0.4. The result showed that 16 participants were needed. By considering potential dropout rates (with an attrition rate of 40%), we recruited 24 participants. The inclusion criteria were as follows: 1) age between 18 and 30 years, and 2) a habit of regular exercise (i.e., aerobic exercise for 30 min once or twice a week). The exclusion criteria were as follows: 1) a history of lower extremity injuries in the preceding 6 months, 2) currently using neuropsychiatric medication, 3) diagnosis of overt neurological diseases, and 4) any contraindications to the use of HD-tDCS (e.g., metal-implanted devices in the brain). Written informed consent was obtained from each participant prior to their participation. The study protocol was approved by the Institutional Review Board of the Shanghai University of Sport (No. 102772022RT050) and conducted according to the Declaration of Helsinki.

### 2.2 Study design

**FIGURE 1:**
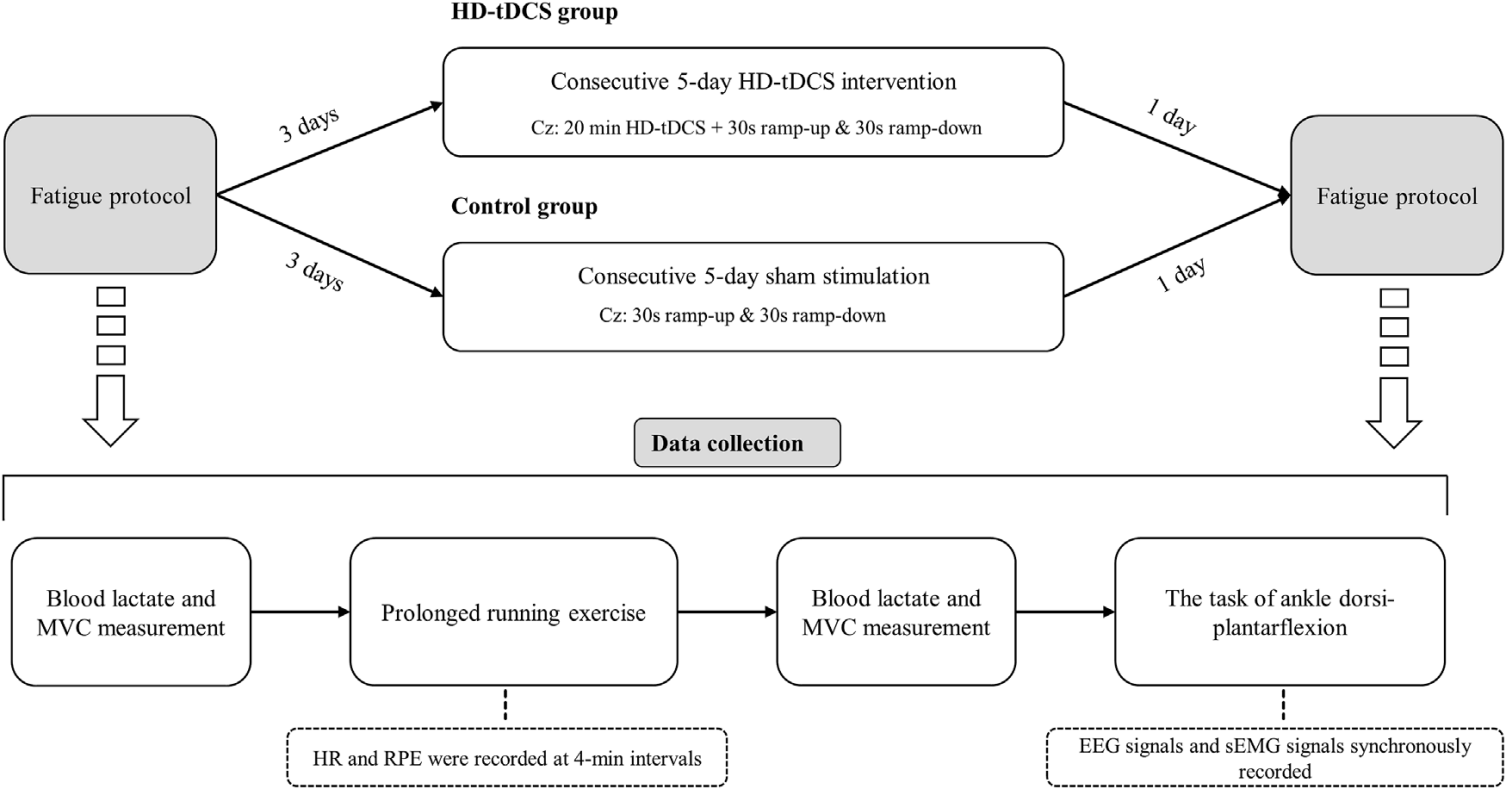
Study design. Participants underwent 5 consecutive days of intervention, in which the participants in the HD-tDCS group received HD-tDCS, and those in the control group received sham stimulation. Three days before and within 1 day after the intervention, the participants completed a prolonged running exercise program. Post-fatigue assessments were completed within 5 min after running to avoid rapid elimination of fatigue.

In this randomized, double-blinded, and sham-controlled parallel study, 24 participants were randomly assigned to HD-tDCS and control groups (*n* = 12 in each group) using computer-generated random numbers. The randomization was conducted by an independent researcher who was not involved in the data collection or analysis. Participants underwent five consecutive days of intervention, in which the participants in the HD-tDCS group received HD-tDCS, and those in the control group received sham stimulation. Multiple-sessions of tDCS induce cumulative and longer-term effects on the functions [e.g., cortical neural plasticity changes (Talsma et al., 2017)]. Previous study showed that five consecutive days of tDCS can induce significant effects on CMFC (Padalino et al., 2021), one of the primary outcomes of our study. We thus designed the protocol here by using five consecutive sessions of tDCS. Three days before and within 1 day after the intervention, the participants completed a prolonged running exercise program (Figure 1). Post-fatigue assessments were completed within 5 min after running to avoid rapid elimination of fatigue. The participants were required to maintain their daily lifestyles throughout the study, avoid vigorous activities for 24 h prior to each exercise session, and abstain from caffeine intake 12 h before each exercise session. Participants were allowed to maintain their regular running habits but were instructed to reduce the intensity and duration of their exercise to avoid inducing fatigue. The running velocity and duration were kept consistent for both the two exercises (i.e., the same fatigue degree across two visits).

### 2.3 Protocol of running-induced fatigue

The participants were instructed to complete a running test on a treadmill (Gymrol S2500, HEF Techmachine, Andrézieux-Bouthéon, France) with a 1% slope to determine individualized constant velocity in prolonged running (Racinais et al., 2007). The velocity was set at 8 km/h for the initial 3 min and then increased with 0.5 km/h per minute until the first ventilatory threshold occurred (Racinais et al., 2007), as measured by gases expired via a wearable metabolic system (K4b2, Cosmed, Rome, Italy) and determined by following an established reference (Davis, 1985). Running velocity corresponding to the occurrence of the first ventilation threshold was then used as individualized constant velocity in prolonged running exercise.

Then, each participant completed one prolonged running exercise on a treadmill at their own constant running velocity before and after the intervention. Heart rate (HR) and rating of perceived exertion (RPE) were recorded every 4 min. Fatigue condition was determined when participants met all of the following criteria: 1) participants cannot maintain the constant running velocity despite strong verbal encouragement, 2) the RPE rating was at least 19, and 3) their HR reached 90% of their maximum HR (i.e., 220 minus age) (Ramos-Campo et al., 2017; Wang et al., 2021). Blood lactate and maximal voluntary isometric contraction (MVC) of ankle dorsiflexors and plantarflexors were measured before and after running-induced fatigue. No significant differences were observed in the individualized constant running velocity (HD-tDCS group vs. control group: 10.7 ± 1.0 km/h vs. 10.5 ± 1.0 km/h, *p* = 0.532) and duration time (HD-tDCS group vs. control group: 34.8 ± 5.6 min vs. 36.1 ± 8.6 min, *p* = 0.678) between the HD-tDCS and control groups.

### 2.4 HD-tDCS protocol

**FIGURE 2:**
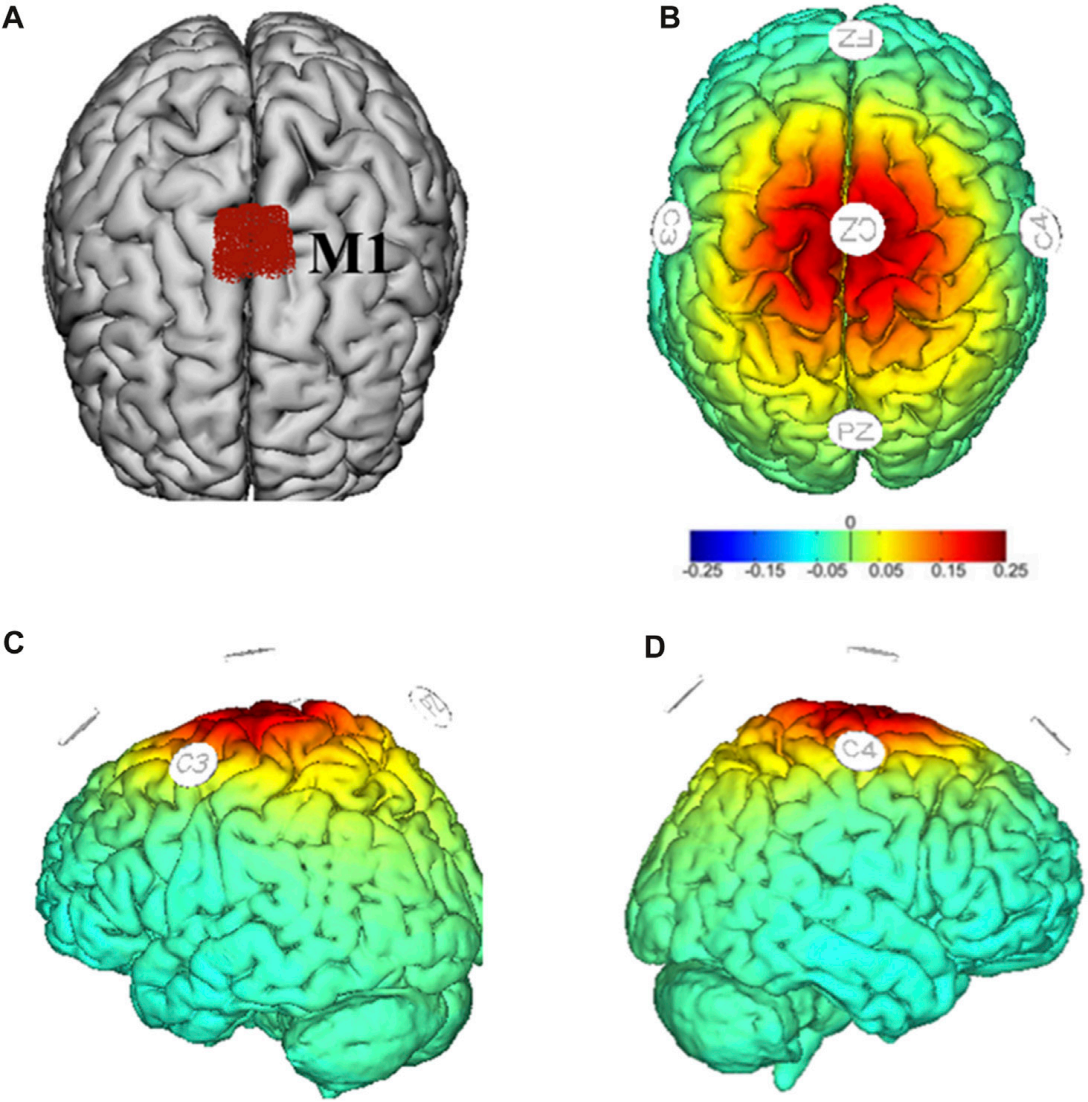
Target region (M1) in red **(A)** and montages of HD-tDCS and simulated electric field distribution **(B–D)**. Warmer colors reflect higher current intensity, while cooler colors indicate lower current intensity.

HD-tDCS was administered by a multichannel NeuroConn^®^ DC-stimulator system (DC-stimulator MC, Neurocare group, Munich, Germany) connected to five ring-type electrodes (3.5 cm^2^): one placed on the primary motor cortex (M1) as an anode electrode (i.e., Cz) and the remaining four placed around it as return electrodes (i.e., C3, C4, Fz, and Pz) based on the international standard 10/20 system (Figure 2) (Xiao et al., 2022). The Cz region usually covers bilateral lower-limb area of M1 (Mizuno and Aramaki, 2017). Studies have shown that tDCS targeting the Cz region can modulate the activity of M1, thereby influencing motor control and coordination (Park et al., 2019; Hou et al., 2022). The HD-tDCS consisted of 20-min stimulation of current intensity of 2 mA (Workman et al., 2020a; Workman et al., 2020b). The current ramped up to maximum intensity in the first 30 s and ramped down to 0 in the last 30 s of the stimulation. The sham stimulation consisted of 60-s current delivery only (ramping up and down 30 s each at the beginning without supplying current in the remaining period (Xiao et al., 2022). The current intensity and duration length of one stimulation session was determined by considering both safety and maximizing the effects of stimulation. Previous studies showed that 2 mA was the maximum current intensity of one electrode that can safely stimulate the brain without causing serious adverse event (Bikson et al., 2016; Buchanan et al., 2023). Another study showed that the effects of tDCS on neural plasticity reached maximum when stimulating for 20 min, and increasing the duration length of one stimulation session beyond 20 min cannot induce greater effects (Mosayebi Samani et al., 2019). Therefore, almost all the studies using tDCS used this kind of design and we here also followed this design. We here used traditional in-active sham that was believed to not induce potential significant effects on the excitability of targeting regions. The device was operated by a trained research assistant who did not participate in any other procedures of the study. The participants and researchers were unaware of the stimulation condition. Blinding efficacy and adverse effects were assessed at the last stimulation by asking the participants to complete questionnaires to subjectively judge the type of stimulation and report if any side effects or uncomfortable feelings were experienced, respectively (Valiengo et al., 2020).

### 2.5 Data collection

#### 2.5.1 Torque measurement

The definition of the dominant foot is determined by the answer of participants to “which leg they prefer to use for kicking a ball” (Fu et al., 2017). MVC torques of the dominant ankle dorsiflexors and plantarflexors were measured with a dynamometer (Con-Trex, Physiomed, Freistaat Bayern, Germany). The participants were placed in prone position with pelvis, knee, and ankle angulations at anatomical zero position and their foot strapped by Velcro straps. The participants were strongly inspired and asked to avoid compensation from other joints during the measurement. MVC torque was determined as the maximal force across three trials, which were separated by a 1 min interval (Spedden et al., 2018). Higher MVC can reflect the muscle’s ability to generate greater force at its maximum, which is beneficial to improve motor control performance (Østerås et al., 2002).

#### 2.5.2 Electrophysiological recordings

Electroencephalography (EEG) and surface electromyography (sEMG) signals were recorded synchronously during the maximal isokinetic (60°/s) task of dominant ankle dorsi–plantarflexion (15° of dorsiflexion and 30° of plantarflexion) at pre- and post-intervention under fatigue. The position of the participants was the same as that in the torque measurement. Four EEG channels (i.e., C1, C2, Cz, and FCz) were selected because of their association with ankle movement (Perez et al., 2006; Spedden et al., 2018). The EEG signal of each channel was recorded using a wireless EEG cap (Nsw364, Neuracle, Changzhou, China) with a sampling rate of 1,000 Hz. The impedance of each EEG electrode was maintained below 4 kΩ. The sEMG signals of the tibialis anterior (TA), medial gastrocnemius (MG), and lateral gastrocnemius (LG) muscles were recorded using a wireless sEMG acquisition system (Noraxon Myomuscle, Noraxon Inc., Arizona, the United States) with a sampling rate of 2,000 Hz. The bipolar electrodes were longitudinally placed in the central part of the muscle with an electrode spacing of 2 cm. Prior to electrode placement, the skin was shaved and cleaned with alcohol to minimize impedance as much as possible. The torque curves during the task were also recorded. A custom hardware device (i.e., a trigger signal generator) and a synchronization cable were used to ensure synchronous acquisition of the torque curves, EEG and sEMG data that were recorded by different computers.

### 2.6 Data processing

EEG and sEMG data were pre-processed and calculated using MATLAB R2018b (MathWorks, Inc., Natick, MA, United States). Channels with data of obvious artifacts were rejected by visual inspection. The outliers were interpolated, and the data were re-referenced to an average reference (Forman et al., 2022). Band-pass filter was applied within the frequency range of 0.5–60 Hz (Lu and Sorooshyari, 2023), and independent component analysis was run to remove noises from the eyes, muscles, and heart.

sEMG data were bandpass filtered within the frequency range of 10–500 Hz. The root mean square (RMS) of each muscle was calculated based on the envelope of sEMG signals (Wang et al., 2022). Higher RMS values represent a higher level of muscle activation. The RMS of each muscle was normalized using the peak RMS amplitude of the respective sEMG signals recorded during the ankle dorsi-plantarflexion after the first prolonged running exercise.

The epochs of dorsiflexion and plantarflexion were separated according to the torque curves collected during the ankle dorsi–plantarflexion task.

The linear coupling between synchronously recorded EEG and sEMG signals was analyzed using CMC (Yang et al., 2009; Siemionow et al., 2010; Tuncel et al., 2010; Xu et al., 2023). We here focused on the coupling in the beta (16–30 Hz) and gamma (31–50 Hz) bands as evidence has shown strong coherence between the EEG and sEMG signals within these bands and were associated with motor control (Chakarov et al., 2009; Yang et al., 2009). Pre-processed EEG and sEMG data from the two epochs of dorsiflexion and plantarflexion movements were used to obtain signals in the beta and gamma bands. Then, the CMC of the EEG data of each channel (C1, C2, Cz, FCz) and the sEMG data of dorsiflexor (i.e., TA) and plantarflexors (i.e., MG and LG) in these two bands were calculated as follows (Mima and Hallett, 1999):

$$C_{xy}=\frac{{\left.{\left|S\right.}_{xy}\left(f\right)\right|}^{2}}{S_{xx}\left(f\right)\times S_{yy}\left(f\right)}$$

$$S_{xy}\left(f\right)=\frac{1}{n}\sum_{i=1}^{n}x_{i}\left(f\right)\times y_{i}{\left(f\right)}^{*}$$

where *x* and *y* represent two types of signals, *S*_*xx*_(*f*) and *S*_*yy*_(*f*) represent the auto-spectra of the signals *x* and *y* at frequency *f*, respectively, and *S*_*xy*_(*f*) represents the cross-spectrum of the signal *x* and *y* at frequency *f*. The CMC value is denoted by [0,1], where 0 represents the lack of coherence between the two signals; values closer to 1 indicate a higher degree of synchronization between the signals. CMC was considered significant if it is above the confidence level (CL), which was calculated as follows (Halliday et al., 1995):

$$CL=1-{\left(1-\alpha \right)}^{\frac{1}{N-1}}$$

where *N* represents the number of data segments, and *α* is the significance level. In this study, the number of data segments was 512, and the significance level was 0.95.

### 2.7 Statistical analysis

Continuous data were presented as mean ± standard deviation (SD). Shapiro–Wilk test was used to test the normality of all continuous data. When the data were normally distributed, one-way ANOVA models were used to assess significant differences in running performance (i.e., individualized constant running velocity and duration time), physiological metrics (i.e., blood lactate, HR), MVC torques, CMC, and RMS between groups (i.e., HD-tDCS and sham) before the intervention. When the data were not normally distributed, non-parametric Friedman test was used. Wilcoxon signed-rank test was performed to assess significant differences in RPE (non-continuous data) between the two groups before the intervention.

To examine the effects of fatigue on the physiological metrics and MVC torques within the first prolonged running exercise, one-way ANOVA models were used when the data were normally distributed. Otherwise, non-parametric Friedman test was implemented. The dependent variables were blood lactate, HR, and MVC torque for each model, and the factor was time (pre-, post-fatigue). Wilcoxon signed-rank test was performed to determine the effects of fatigue on RPE in the first prolonged running exercise.

To examine the effects of HD-tDCS on CMC and muscular activities of dorsiflexors and plantarflexors under fatigue, two-way repeated-measures ANOVA models adjusted for age, height, and weight were used when the data were normally distributed. To note, if there is a significant difference in any outcomes before the intervention between the two groups, we included the pre-intervention outcomes into the model. The dependent variables were CMC, RMS, and MVC torque for each model. Model factors were stimulation condition (HD-tDCS, sham), time (pre-, post-intervention), and their interaction. Bonferroni’s test was used for *post hoc* analysis to determine where the significance was in the ANOVA models. Partial eta-square ( $\left.\eta_{p}^{2}\right)$ and Cohen’s *d* were reported to the ANOVA models and *post hoc* analyses as effect size values, respectively. When the data were not normally distributed, non-parametric Friedman test was used.

We further explored the relationship between CMC and muscular activities of dorsiflexors and plantarflexors by using Pearson’s correlation analyses. The association between these outcomes before the intervention, as well as between the percent changes of those with significant changes induced by HD-tDCS, was examined.

All statistical analyses were performed using SPSS 21.0 Software (SPSS Inc., Chicago, IL, the United States). The significance level was set at *p* < 0.05.

## 3 Results

All participants successfully completed the assessments. No significant differences were observed in age, height, and weight between the two groups (*p* > 0.492, Table 1). No side effects of HD-tDCS were reported. For blinding efficacy, a 52.7% error rate in guessing the stimulation condition was observed, suggesting successful blinding. All the continuous data were normally distributed. No significant differences were observed between the two groups at baseline (*p* > 0.103). Pearson’s correlation analyses revealed positive correlations between the RMS of TA muscle and the CMC between M1 and TA muscle in both the beta (C1-TA: *r* = 0.579, *p* = 0.003; Cz-TA: *r* = 0.573, *p* = 0.003; C2-TA: *r* = 0.619, *p* = 0.001; FCz-TA: *r* = 0.597, *p* = 0.002) and gamma (C1-TA: *r* = 0.460, *p* = 0.024; Cz-TA: *r* = 0.542, *p* = 0.006; C2-TA: *r* = 0.479, *p* = 0.018; FCz-TA: *r* = 0.583, *p* = 0.003) bands at baseline across all participants. No other correlations between CMC and muscular activities of dorsiflexors and plantarflexors were observed (*p* > 0.163).

**TABLE 1:** Demographic information of participants.

Groups	Age (years)	Height (cm)	Weight (kg)
HD-tDCS group (*n* = 12)	21.5 ± 2.2	176.3 ± 5.0	69.3 ± 7.0
Control group (*n* = 12)	21.7 ± 2.3	175.0 ± 7.4	68.8 ± 8.3

### 3.1 Effects of running-induced fatigue on MVC torque and physiological metrics

**FIGURE 3:**
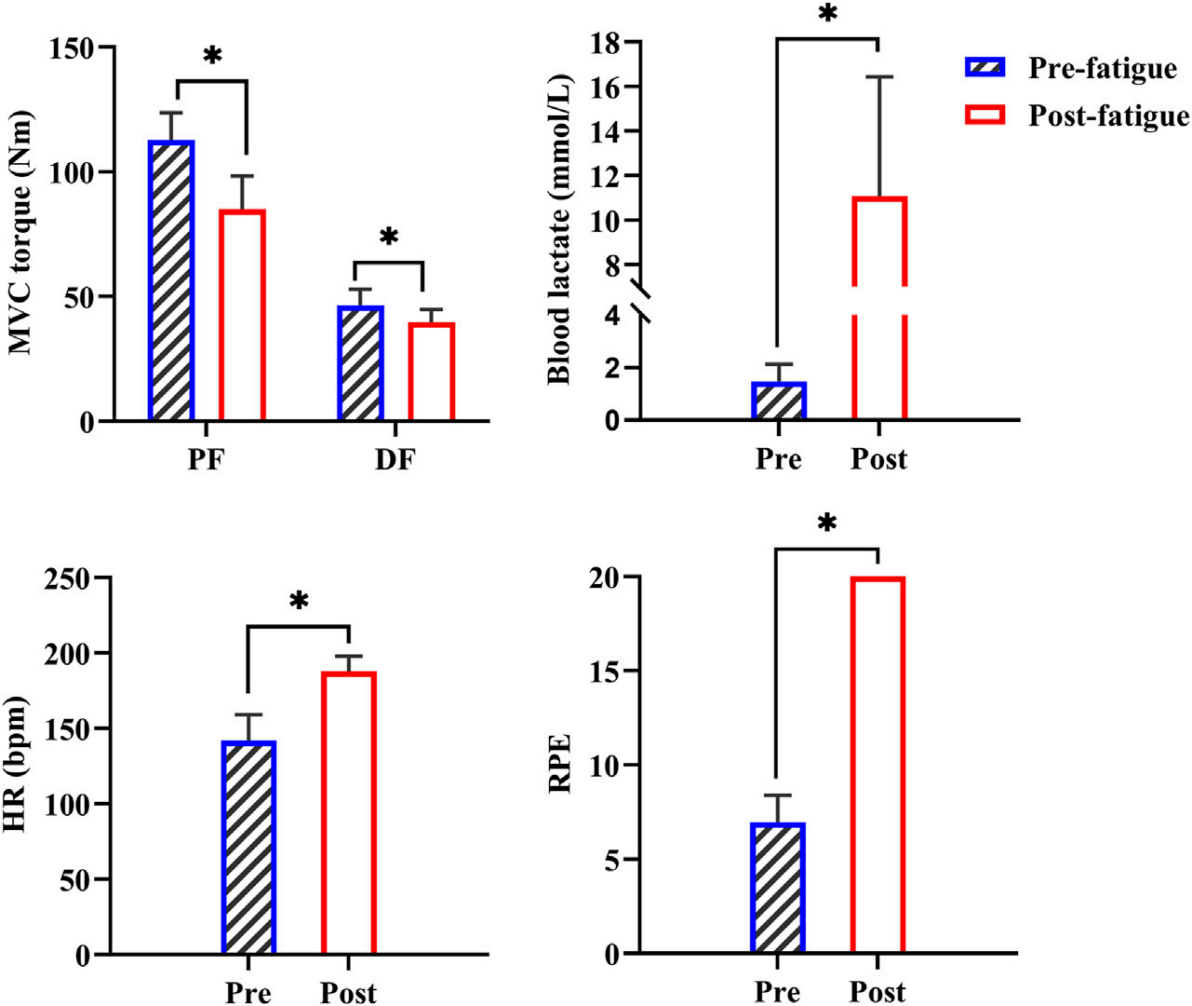
Changes in MVC torques and physiological metrics before and after the first prolonged running exercise. PF: plantarflexors; DF: dorsiflexors; *: *p* < 0.05.

The one-way ANOVA models showed that the MVC torques of dorsiflexors (*p* < 0.001) and plantarflexors (*p* < 0.001) significantly decreased and the blood lactate (*p* < 0.001), HR (*p* < 0.001), and RPE (*p* < 0.001) significantly increased after fatigue compared with those before fatigue. This finding suggests that the prolonged running exercise successfully caused fatigue (Figure 3).

### 3.2 Effect of HD-tDCS on CMC

**FIGURE 4:**
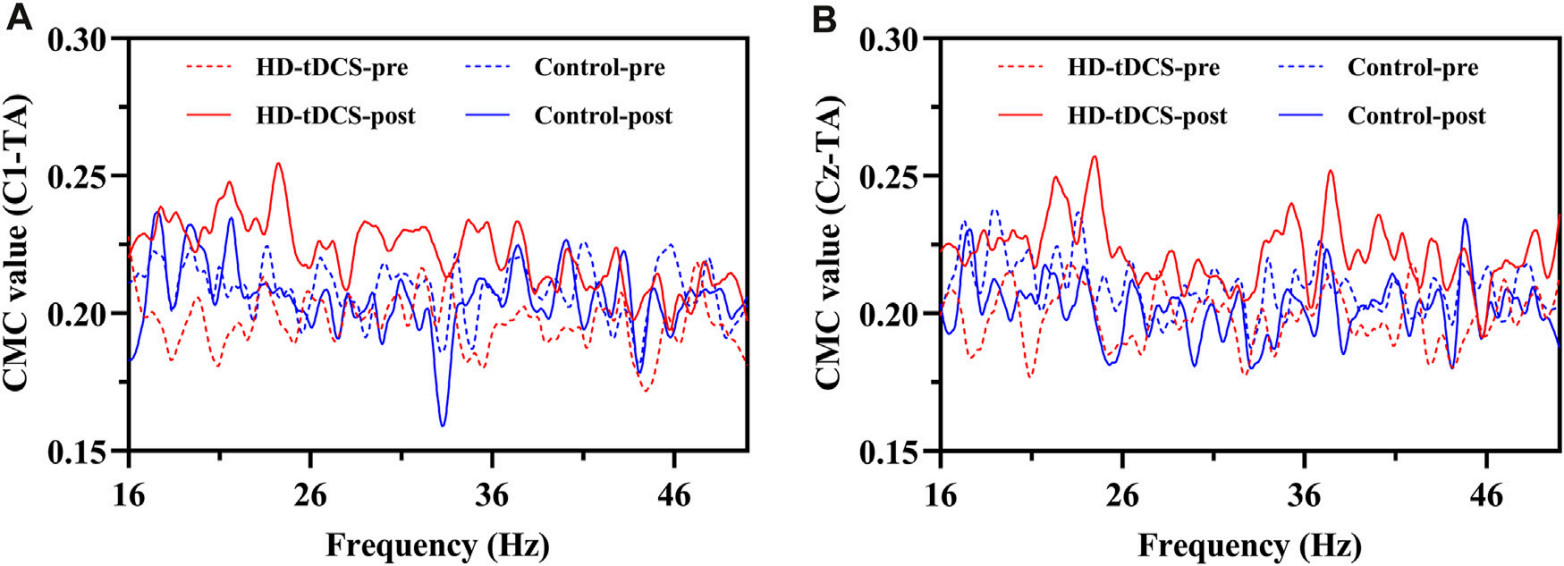
The curves of mean CMC in the beta (16–30 Hz) and gamma (31–50 Hz) bands between the EEG signals of C1 **(A)** and Cz **(B)** and the sEMG signal of TA under fatigue in HD-tDCS and control groups at pre- and post-intervention.

In the beta band (Table 2), two-way repeated-measures ANOVA models indicated significant interactions between stimulation condition and time on C1-TA (*F*_[1, 22]_ = 6.943, *p* = 0.015, $\eta_{p}^{2}=0.240$ ) and Cz-TA (*F*_[1, 22]_ = 6.301, *p* = 0.020, $\eta_{p}^{2}=0.223$ ). *Post-hoc* analyses revealed that the CMC values between the EEG signals of C1 and Cz and the sEMG signal of TA significantly increased after the HD-tDCS intervention compared with those at pre-intervention (C1-TA: *p* = 0.011, Cohen’s *d* = 0.911, Figure 4A; Cz-TA: *p* = 0.032, Cohen’s *d* = 0.767; Figure 4B) in the HD-tDCS group and post-intervention (C1-TA: *p* = 0.038, Cohen’s *d* = 0.901; Cz-TA: *p* = 0.045, Cohen’s *d* = 0.866) in the control group.

**TABLE 2:** CMC of EEG-sEMG in the beta and gamma bands under fatigue.

Beta band (16–30 Hz)							Gamma band (31–50 Hz)				
(Mean ± SD)		HD-tDCS group		Control group		*p*-value	HD-tDCS group		Control group		*p*-value
EEG	sEMG	Pre	Post	Pre	Post		Pre	Post	Pre	Post	
C1											
—	TA	0.206 ± 0.029^A^	0.236 ± 0.035^B^	0.216 ± 0.027^AB^	0.206 ± 0.032^A^	**0.015**	0.206 ± 0.030^A^	0.230 ± 0.038^B^	0.211 ± 0.028^AB^	0.199 ± 0.034^A^	**0.014**
—	MG	0.218 ± 0.028	0.213 ± 0.028	0.231 ± 0.029	0.221 ± 0.034	0.605	0.228 ± 0.039	0.216 ± 0.029	0.228 ± 0.027	0.212 ± 0.034	0.683
—	LG	0.220 ± 0.031	0.216 ± 0.031	0.232 ± 0.035	0.225 ± 0.038	0.746	0.227 ± 0.028	0.222 ± 0.032	0.232 ± 0.033	0.216 ± 0.032	0.212
C2											
—	TA	0.208 ± 0.027	0.226 ± 0.033	0.219 ± 0.028	0.210 ± 0.036	0.121	0.205 ± 0.021	0.220 ± 0.030	0.212 ± 0.027	0.204 ± 0.030	0.104
—	MG	0.218 ± 0.028	0.217 ± 0.028	0.233 ± 0.028	0.218 ± 0.035	0.259	0.221 ± 0.033	0.219 ± 0.037	0.226 ± 0.026	0.216 ± 0.033	0.520
—	LG	0.220 ± 0.030	0.215 ± 0.028	0.226 ± 0.028	0.217 ± 0.033	0.769	0.219 ± 0.032	0.215 ± 0.033	0.226 ± 0.031	0.217 ± 0.030	0.573
Cz											
—	TA	0.207 ± 0.030^A^	0.233 ± 0.037^B^	0.218 ± 0.028^AB^	0.204 ± 0.029^A^	**0.020**	0.200 ± 0.024^A^	0.226 ± 0.030^B^	0.211 ± 0.028^AB^	0.201 ± 0.029^A^	**0.022**
—	MG	0.219 ± 0.034	0.213 ± 0.031	0.230 ± 0.030	0.218 ± 0.030	0.645	0.227 ± 0.038	0.217 ± 0.033	0.228 ± 0.026	0.214 ± 0.031	0.706
—	LG	0.220 ± 0.034	0.212 ± 0.032	0.227 ± 0.031	0.217 ± 0.030	0.834	0.226 ± 0.035	0.216 ± 0.032	0.232 ± 0.031	0.215 ± 0.032	0.498
FCz											
—	TA	0.206 ± 0.026	0.227 ± 0.037	0.218 ± 0.034	0.203 ± 0.037	0.055	0.204 ± 0.026	0.222 ± 0.035	0.215 ± 0.027	0.202 ± 0.034	0.077
—	MG	0.218 ± 0.034	0.216 ± 0.035	0.228 ± 0.032	0.218 ± 0.032	0.385	0.222 ± 0.036	0.223 ± 0.038	0.225 ± 0.031	0.215 ± 0.036	0.345
—	LG	0.218 ± 0.029	0.214 ± 0.036	0.225 ± 0.031	0.221 ± 0.035	0.963	0.223 ± 0.031	0.220 ± 0.027	0.228 ± 0.033	0.215 ± 0.032	0.305

Notes: *p*-value: stimulation condition and time interaction effect; different superscript letters (A and B) in each row denote which specific groups or times are significantly different from one another as determined by *post hoc* analyses of ANOVA, models with a significant interaction term between stimulation condition and time. However, as long as the same superscript letter exists, it indicates that there is no significant difference.

The bold values represent the presence of a significant interaction effect between stimulation condition and time.

In the gamma band (Table 2), the ANOVA models revealed significant interactions between stimulation condition and time on C1-TA (*F*_[1, 22]_ = 7.067, *p* = 0.014, $\eta_{p}^{2}=0.243$ ) and Cz-TA (*F*_[1, 22]_ = 6.103, *p* = 0.022, $\eta_{p}^{2}=0.217$ ). *Post-hoc* analyses revealed that the CMC values between the EEG signals of C1 and Cz and the sEMG signal of TA significantly increased after the HD-tDCS intervention compared with those at pre-intervention (C1-TA: *p* = 0.023, Cohen’s *d* = 0.700, Figure 4A; Cz-TA: *p* = 0.022, Cohen’s *d* = 0.938; Figure 4B) in the HD-tDCS group and post-intervention (C1-TA: *p* = 0.046, Cohen’s *d* = 0.863; Cz-TA: *p* = 0.045, Cohen’s *d* = 0.866) in the control group. Additionally, significant main effects of time were observed on Cz-MG (*F*_[1, 22]_ = 5.867, *p* = 0.024, $\eta_{p}^{2}=0.211$ ), C1-LG (*F*_[1, 22]_ = 5.565, *p* = 0.028, $\eta_{p}^{2}=0.202$ ), and Cz-LG (*F*_[1, 22]_ = 7.654, *p* = 0.011, $\eta_{p}^{2}=0.258$ ). This finding indicates a significant decrease from the pre- to the post-intervention values regardless of the group. The other metrics tested had no significant differences (*p* > 0.055).

### 3.3 Effects of HD-tDCS on RMS and MVC torque

The two-way repeated-measures ANOVA models indicated a significant interaction between stimulation condition and time on the RMS value of TA (*F*_[1, 22]_ = 9.820, *p* = 0.005, $\eta_{p}^{2}=0.309$ , Table 3). *Post-hoc* analysis indicated that the RMS value of TA significantly increased after the HD-tDCS intervention compared with that at pre-intervention (*p* = 0.022, Cohen’s *d* = 0.757) in the HD-tDCS group and post-intervention (*p* = 0.042, Cohen’s *d* = 0.880) in the control group. Additionally, significant main effects of time were observed on the RMS values of LG (*F*_[1, 22]_ = 16.377, *p* = 0.001, $\eta_{p}^{2}=0.427$ ) and MG (*F*_[1, 22]_ = 4.619, *p* = 0.043, $\eta_{p}^{2}=0.174$ ), reflecting a significant decrease from the pre- to the post-intervention values regardless of the group.

**TABLE 3:** MVC torque and RMS under fatigue at pre- and post-intervention.

(Mean ± SD)		HD-tDCS group		Control group		
		Pre	Post	Pre	Post	*p*-value
RMS, %	TA	69.34 ± 4.51^A^	73.51 ± 6.36^B^	71.12 ± 3.87^AB^	67.77 ± 6.71^A^	**0.005**
	LG	69.16 ± 4.81	61.48 ± 6.89	66.71 ± 5.63	62.69 ± 6.75	0.218
	MG	69.25 ± 7.13	64.90 ± 4.11	67.70 ± 5.21	65.65 ± 5.71	0.450
MVC, Nm	Dorsiflexors	41.48 ± 5.57^A^	45.07 ± 7.96^B^	38.14 ± 4.42^AB^	36.40 ± 3.72^A^	**0.014**
	Plantarflexors	82.11 ± 12.76	82.28 ± 15.79	88.11 ± 13.39	82.63 ± 21.42	0.560

Notes: *p*-value: stimulation condition and time interaction effect; different superscript letters (A and B) in each row denote which specific groups or times are significantly different from one another as determined by *post hoc* analyses of ANOVA, models with a significant interaction term between stimulation condition and time. However, as long as the same superscript letter exists, it indicates that there is no significant difference.

The bold values represent the presence of a significant interaction effect between stimulation condition and time.

The ANOVA models revealed a significant interaction (*F*_[1, 22]_ = 7.169, *p* = 0.014, $\eta_{p}^{2}=0.246$ ) between stimulation condition and time on the MVC torque of dorsiflexors (Table 3). *Post-hoc* analysis revealed that the MVC torque of dorsiflexors significantly increased after the HD-tDCS intervention compared with that at pre-intervention (*p* = 0.018, Cohen’s *d* = 0.522**)** in the HD-tDCS group and post-intervention (*p* = 0.002, Cohen’s *d* = 1.395) in the control group. A main effect (*F*_[1, 22]_ = 8.314, *p* = 0.009, $\eta_{p}^{2}=0.274$ ) of stimulation condition was also observed. Additionally, within the HD-tDCS group, no significant correlation appeared between the percent changes in CMFC and muscular activities of dorsiflexors and plantarflexors after the intervention (*p* > 0.308).

## 4 Discussion

To the best of our knowledge, this study is the first to demonstrate the evidence that HD-tDCS can significantly increase the CMC between TA and the motor cortex in the beta and gamma bands under running-induced fatigue, and the higher CMC values occurred in the midline and contralateral brain areas of TA, suggesting that HD-tDCS targeting M1 improved CMFC. These results suggested that HD-tDCS applied over M1 holds great promise to enhance the motor control performance of lower extremities and enhance the CMFC under fatigue, providing critical knowledge for the design of future studies and rehabilitative programs to alleviate the influences of exercise-induced fatigue on motor control.

HD-tDCS significantly improved the beta- and gamma-band CMC between the EEG signals of C1 and Cz and the sEMG signal of TA. This finding is consistent with previous reports on the effect of HD-tDCS on CMC under non-fatigue conditions (Bao et al., 2019; Chen et al., 2019). For example, Bao et al. (Bao et al., 2019) observed that anodal HD-tDCS significantly facilitated CMFC compared with cathode and sham stimulation, as assessed by increased CMC, in medium-level isometric wrist extension tasks. TA is the muscle maintaining high-level activation during running, making it more susceptible to fatigue than other lower extremity muscles (Reber et al., 1993). Afferent feedback from fatigued muscles can inhibit neural drives at the spinal and supraspinal levels through afferent fibers in types Ⅲ and Ⅳ (Millet and Lepers, 2004; Taylor et al., 2006), potentially altering the top–down (i.e., from central to peripheral) regulation of motor control under fatigue (Nicol et al., 2006; Millet et al., 2018). The observed improvement in functional coupling between TA and motor cortex induced by tDCS may arise from the increased excitability of M1, thereby increasing the neural drive in the descending corticospinal pathway (Dutta and Chugh, 2011). Additionally, previous studies showed that dynamic exercise-induced fatigue (e.g., running) can significantly change CMC in the beta and gamma bands (Yang et al., 2009; Tuncel et al., 2010; Xu et al., 2023). These bands are closely associated with the maintenance of static muscle power output (Conway et al., 1995) and help integrate visual and proprioceptive information for appropriate command of motor control (Omlor et al., 2007). Therefore, the significant improvement in CMC in the beta and gamma bands induced by HD-tDCS may be beneficial for the static motor control of TA in the coronal plane (i.e., inversion) of ankle joint and for the maintenance of dorsiflexion angle during running to prevent ankle sprain caused by over-inversion under fatigue.

We observed that HD-tDCS can significantly increase the muscular activities of dorsiflexors (i.e., MVC torque and RMS of TA) under running-induced fatigue. These observations are in line with the findings that tDCS targeting M1 improved muscular endurance performance (Tanaka et al., 2009; Lu et al., 2021; Wang et al., 2022). Still, the effects of tDCS on muscle activity during fatiguing exercise are a topic of controversy, particularly among young and healthy participants. For example, Workman et al., (Workman et al., 2020b), observed that single-session of tDCS targeting the dominant M1 increased the fatigability in the knee extensor of young healthy participants, adversely affecting fatigue performance. One potential reason for the inconsistent observations for the effects of tDCS on fatigue may be due to the different types of exercises inducing fatigue. Compared to single-joint movement tasks, whole-body exercise tasks (e.g., running exercise we used here) may require more neural activation and coordination, making it more likely to induce fatigue in supraspinal level (Jubeau et al., 2014). Therefore, tDCS targeting the supraspinal regions may induce greater effects on fatigue related to this type of exercise. Fatigue can disturb the balance between the excitation and inhibition of the motoneuron pool (Racinais et al., 2007; Girard et al., 2011), inhibit the response of spinal motoneurons (Andersen et al., 2003), and affect communication at the neuromuscular junction, resulting in decreased muscle force output (Millet and Lepers, 2004). One of the potential underlying mechanisms through which tDCS enhances muscular activity may be the increased excitability of M1 induced by tDCS because it can improve the efficiency of cortical processing (Wang et al., 2022) and the recruitment of motor units (Lu et al., 2021). The increased motor unit recruitment is directly related to increased motoneuron pool excitability, and thus the enhanced CMFC (Abdelmoula et al., 2016). Additionally, the rate of force loss can be reduced by increasing the cortical excitability (Cogiamanian et al., 2007). Hence, improved muscle activation and increased muscle strength induced by HD-tDCS may be related to the increased excitability of M1 and enhanced CMFC, which potentially helps improve the excitability of the spinal motoneuron pool and reduce the loss of force. We did not observe any significant associations between the percent change in CMFC and the muscular activities of ankle dorsi–plantarflexion after HD-tDCS intervention, which could be due to the small sample size. The association between the HD-tDCS-induced changes in CMFC and that in muscular activities is thus highly-demanded to be more explicitly examined in future studies with larger sample sizes.

We acknowledge that there may be potential placebo effects of sham stimulation as induced by subjective confidence on the stimulation. Therefore, it is worthwhile to more explicitly assess the subjective confidence of participants on the stimulation type, enabling the examination of such potential placebo effects. Additionally, in the current literature, the neurobiological effects of sham stimulation remain incompletely elucidated. Future studies should also focus on optimizing sham stimulation protocols to mitigate their potential placebo effects.

Fatigue is somehow a protective mechanism in the human body. However, we believe the effects of tDCS on fatigue do not arise from the inhibition of the detection of fatigue of the underlying neurophysiological procedure, which may be harmful to humans, but instead, by enhancing the endurance capacity, alertness, and attention (Hanken et al., 2016). We thus believe this kind of improvement may not be harmful to individuals. Still, we agree that studies examining those underlying elements related to fatigue is highly-demanded to advance our understanding of such tDCS-induced benefits for fatigue.

Several limitations in this study should be noted. First, a small sample size of male recreational young adults was recruited, and only short-term effects of HD-tDCS were examined. Future studies with larger sample sizes, matched numbers of men and women, and longer periods of follow-up assessments are thus needed to confirm the observations here. Second, we used group-based HD-tDCS montage. The differences in the brain anatomy and brain connectivity patterns among individuals may interfere with the effects of HD-tDCS. Individualized HD-tDCS montage should be implemented in future studies to maximize its effects.

## 5 Conclusion

This study demonstrates that HD-tDCS can significantly increase the corticomuscular coherence between TA and the motor cortex and the muscle activation of dorsiflexors under running-induced fatigue, suggesting that HD-tDCS targeting M1 improved cortical–muscular functional coupling and muscular activities of ankle dorsiflexion under the influence of running-induced fatigue.

## Data Availability

The raw data supporting the conclusion of this article will be made available by the authors, without undue reservation.
